# HIF-1α in ischemic stroke: context-dependent roles in ferroptosis and neurovascular repair

**DOI:** 10.3389/fneur.2026.1876838

**Published:** 2026-09-11

**Authors:** Mao-Mei Song, Jian-Ming Wang, Xiao-Feng Li, Chang-Xin Li, Sheng-Qin Yao, Jun-Ying Wu, Shi-Na Song

**Affiliations:** 1 Department of Neurology, Headache Center, The First Hospital of Shanxi Medical University, Taiyuan, Shanxi, China; 2 Department of Aesthetic Plastic Surgery, Shanxi Provincial People’s Hospital, Taiyuan, Shanxi, China; 3 Department of General Medicine, Linfen City People’s Hospital, Linfen, Shanxi, China; 4 Department of General Surgery, Third Hospital of Shanxi Medical University, Taiyuan, Shanxi, China; 5 Department of Rehabilitation, The First Hospital of Shanxi Medical University, Taiyuan, Shanxi, China

**Keywords:** angiogenesis, blood–brain barrier, ferroptosis, HIF-1α, ischemic stroke, neurogenesis, neurovascular repair

## Abstract

Stroke remains a leading cause of death and long-term disability worldwide, and effective strategies to limit ischemic injury and promote post-stroke tissue repair remain urgently needed. Ferroptosis, an iron-dependent form of regulated cell death characterized by iron accumulation and lipid peroxidation, has emerged as an important mechanism contributing to neuronal and neurovascular injury after ischemic stroke. Hypoxia-inducible factor-1α (HIF-1α), a central regulator of cellular responses to hypoxia, is closely involved in both ferroptotic injury and post-stroke neurovascular repair. In this review, we summarize current evidence on the context-dependent role of HIF-1α in ferroptosis and neurovascular repair after ischemic stroke. HIF-1α regulates ferroptosis through multiple mechanisms, including modulation of iron homeostasis, antioxidant defense, lipid metabolism, and lipid peroxidation. However, its effects are not uniformly protective or detrimental and may vary according to the severity and duration of ischemia, the timing of HIF-1α activation, the metabolic state and cellular context. Beyond ferroptosis, HIF-1α contributes to post-stroke neurovascular repair by regulating angiogenesis and vascular remodeling, blood–brain barrier restoration, neurogenesis, and neuronal remodeling. These findings suggest that HIF-1α may serve as a molecular link between ischemic injury, ferroptosis, and endogenous repair responses. Rather than simply activating or inhibiting HIF-1α, therapeutic strategies that selectively modulate HIF-1α signaling according to the stage and pathological context of ischemic stroke may provide a more rational approach to limiting ferroptotic injury while promoting neurovascular repair.

## 1 Introduction

Stroke is one of the leading causes of mortality and long-term disability worldwide, with ischemic stroke accounting for the majority of cases. Its pathophysiology is highly complex, involving multiple interconnected processes such as cerebral ischemia and hypoxia, inflammatory responses, and oxidative stress (1). Following stroke, the onset and progression of neuronal injury profoundly impair neurological recovery, making it a central focus in contemporary neurorehabilitation research. Accumulating evidence indicates that ischemia–reperfusion injury not only induces direct neuronal death but also triggers intricate neuroimmune interactions that influence neural circuit remodeling and functional restoration (2). Therefore, a comprehensive understanding of the pathological mechanisms underlying stroke, particularly the diverse forms of cell death and their regulatory pathways, is essential for the development of effective neuroprotective and neurorestorative strategies.

In recent years, ferroptosis has been recognized as a distinct form of regulated cell death characterized by iron dependency and lipid peroxidation, and it has increasingly become a focal point in stroke research (3). Unlike classical forms of cell death such as apoptosis and necrosis, ferroptosis is defined by intracellular iron overload and the accumulation of lipid peroxidation products, ultimately leading to membrane damage and loss of cellular function. Increasing evidence suggests that ferroptosis plays a pivotal role in ischemia–reperfusion injury following stroke, where it contributes to neuroinflammation and neuronal death, exacerbating cerebral tissue damage (4, 5). For instance, iron overload triggers the Fenton reaction during cerebral ischemia–reperfusion, generating excessive free radicals that further induce lipid peroxidation, thus establishing the molecular basis of ferroptosis. Moreover, the occurrence of ferroptosis is closely associated with impairment of antioxidant-based defenses in the nervous system, particularly when the function of glutathione peroxidase 4 (GPX4) is compromised, which markedly increases susceptibility to ferroptosis (6). Accordingly, targeting ferroptosis-related pathways to inhibit iron accumulation and lipid peroxidation has emerged as a promising strategy for neuroprotection after stroke.

Hypoxia-inducible factor-1α (HIF-1α) is a central regulator of the cellular response to hypoxia and plays a crucial role in the hypoxic adaptation that occurs following stroke. HIF-1α modulates cellular metabolism, oxidative stress responses, and cell survival, thereby facilitating adaptive regulation within ischemic regions. Evidence indicates that HIF-1α not only participates in the regulation of angiogenesis and energy metabolism, but may also indirectly influence the occurrence of ferroptosis through the modulation of iron metabolism and the expression of antioxidant genes (7). In addition, multiple signaling pathways governed by HIF-1α promote the proliferation and migration of neural stem cells (NSCs), providing endogenous support for neural repair after stroke (8, 9). Therefore, elucidating the role of HIF-1α in the regulation of ferroptosis and endogenous recovery mechanisms will not only advance our understanding of the pathophysiological mechanisms underlying post-stroke neurorehabilitation, but also provide both theoretical foundations and practical guidance for the development of novel therapeutic strategies.

## 2 HIF-1α in stroke: biological functions and context-dependent roles

### 2.1 The structure and function of HIF-1α

HIF-1α is a key transcription factor that mediates cellular adaptive responses to hypoxic conditions. It primarily promotes metabolic reprogramming, angiogenesis and cell survival under low-oxygen conditions through the regulation of a broad range of target genes (10). Structurally, the HIF-1α protein contains an oxygen-dependent degradation domain (ODD) and a transactivation domain (TAD). The ODD domain is responsible for sensing oxygen availability and regulating the stability of the molecule, whereas the TAD domain is involved in activating the transcription of downstream genes (11). Under normoxic conditions, HIF-1α is readily hydroxylated and subsequently degraded via the ubiquitin-proteasome pathway, resulting in low intracellular levels of protein (12). In contrast, under hypoxic conditions, hydroxylation is inhibited, allowing HIF-1α to accumulate in the cytoplasm before translocating into the nucleus. There, it forms a heterodimer with HIF-1β and subsequently binds to hypoxia-responsive elements within the promoters of target genes. This interaction activates the transcription of numerous genes, including those encoding glycolytic enzymes and vascular endothelial growth factor (VEGF), thereby regulating cellular oxygen metabolism and angiogenesis and promoting cell survival (13). In addition, HIF-1α mitigates oxidative stress and helps maintain cellular homeostasis by modulating the expression of metabolic and antioxidant enzymes (14).

### 2.2 Stage- and context-dependent effects of HIF-1α in ischemic stroke

**Figure 1:**
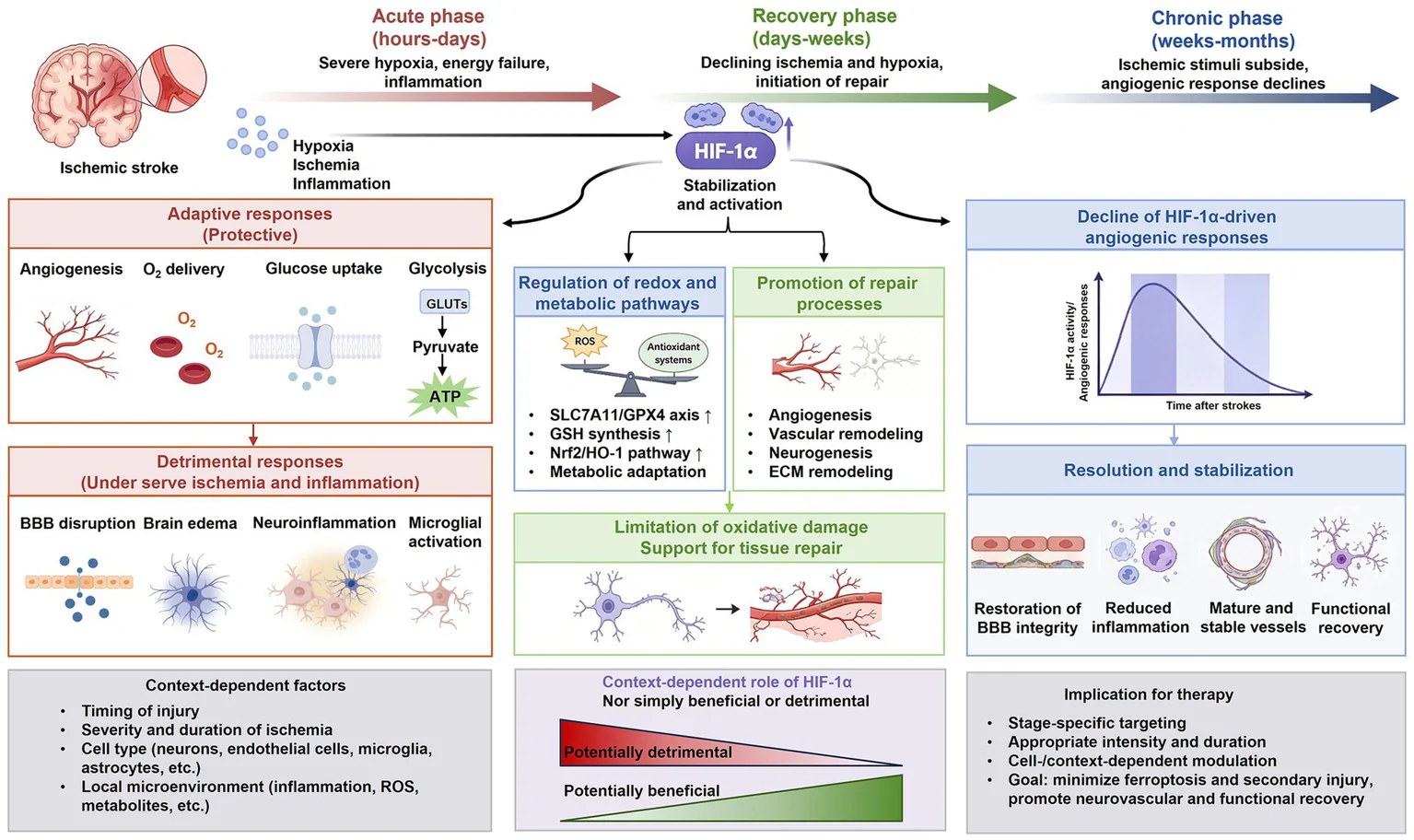
Context-dependent effects of HIF-1α after ischemic stroke. Its activation may promote adaptive responses and tissue repair, while excessive or sustained activation under severe ischemic conditions may contribute to secondary injury.

The effects of HIF-1α after ischemic stroke vary according to the stage of injury and the surrounding pathological microenvironment (15, 16) (Figure 1). During the acute phase, severe hypoxia and energy failure stabilize HIF-1α and activate a range of adaptive responses. HIF-1α helps cells adapt to hypoxic conditions and maintain energy and ionic homeostasis by promoting angiogenesis, oxygen delivery, glucose uptake, and glycolysis (17, 18). However, under conditions of severe ischemia and inflammation, excessive or sustained HIF-1α activation may also have detrimental effects by contributing to blood–brain barrier (BBB) disruption, brain edema, neuroinflammation, and microglial activation, thereby exacerbating secondary brain injury (14, 19). During the chronic or recovery phase, HIF-1α appears to play a more prominent role in maintaining redox homeostasis and promoting tissue repair. It can regulate antioxidant and metabolic pathways to limit reactive oxide species (ROS) accumulation and oxidative damage, while also contributing to post-ischemic angiogenesis and vascular remodeling to support the repair of injured brain tissue (20, 21). As ischemic and hypoxic stimuli gradually subside, HIF-1α-driven angiogenic responses also decline, further highlighting the temporal nature of its effects (16). Thus, the role of HIF-1α after stroke cannot be simply classified as beneficial or detrimental; rather, its effects depend on the timing, severity of ischemic injury, cellular context, and local microenvironment. This context-dependent nature is particularly important when considering the role of HIF-1α in ferroptosis and post-stroke neurovascular repair.

## 3 Ferroptosis in ischemic stroke

### 3.1 Iron metabolism and iron accumulation

Iron metabolism is central to the regulation of ferroptosis, as iron plays dual roles in cellular physiology: it is essential for oxygen transport, DNA synthesis, and enzymatic reactions, yet its dysregulation drives pathological lipid peroxidation (22). Following ischemic stroke, ischemia, oxidative stress, BBB dysfunction, and tissue damage disrupt iron homeostasis, leading to an increase in the intracellular labile iron pool (LIP) and creating a favorable environment for ferroptosis (23). Iron uptake is primarily mediated by transferrin receptor 1 (TfR1), which facilitates the cellular uptake of transferrin-bound iron. Following internalization, iron is released and reduced to Fe^2+^, which can enter the cytosolic LIP, with divalent metal transporter 1 (DMT1) also contributing to intracellular Fe^2+^ transport (24). In contrast, ferroportin (FPN) is the major cellular iron exporter and plays a central role in maintaining intracellular iron balance (25). Following ischemic stroke, increased iron uptake together with impaired iron export may promote iron accumulation in the ischemic brain (26, 27). In addition, BBB disruption after ischemic stroke may further aggravate cerebral iron accumulation. Damage to the BBB can increase the entry of circulating iron-containing components into the brain parenchyma, while hemorrhagic transformation and the degradation of heme- and hemoglobin-derived products may provide additional sources of iron (28, 29). The accumulation of labile Fe^2+^ is particularly important because it can catalyze Fenton reactions and generate highly ROS, linking iron dysregulation to oxidative stress and lipid peroxidation (30).

### 3.2 Lipid metabolism and lipid peroxidation

Lipid peroxidation is the central biochemical process that ultimately drives ferroptotic cell death (31). In ischemic stroke, disturbances in cellular metabolism and membrane lipid remodeling increase the availability of polyunsaturated fatty acid (PUFA)-containing phospholipids, which are particularly susceptible to oxidative attack. When combined with increased ROS and labile iron, these changes promote the accumulation of lipid peroxides and progressively compromise membrane integrity (32, 33). Acyl-CoA synthetase long-chain family member 4 (ACSL4) is a key regulator of ferroptosis-associated lipid remodeling. ACSL4 facilitates the activation of long-chain PUFAs and promotes their incorporation into membrane phospholipids. Lysophosphatidylcholine acyltransferase 3 (LPCAT3) further contributes to the incorporation of PUFA-containing fatty acids into phospholipids, increasing the pool of membrane lipids that are vulnerable to peroxidation. Consequently, increased ACSL4 and LPCAT3 activity can enhance the susceptibility of ischemic cells to ferroptosis by enriching cellular membranes with peroxidation-prone PUFA-containing phospholipids (34, 35). Following ischemic injury, ROS generated from mitochondrial dysfunction, iron-dependent reactions, and inflammatory processes can initiate the oxidation of PUFA-containing phospholipids. Iron-dependent Fenton chemistry further accelerates this process, while lipid-peroxidizing enzymes can contribute to the enzymatic oxidation of PUFA-containing phospholipids. The resulting lipid ROS and lipid hydroperoxides accumulate when cellular antioxidant systems cannot adequately remove them (36–38). Lipid peroxidation products can directly disrupt the structure and function of cellular membranes and organelles. The accumulation of lipid peroxides alters membrane fluidity and permeability and can damage mitochondrial and other intracellular membranes. Secondary lipid-derived reactive aldehydes, including malondialdehyde and 4-hydroxynonenal, can further modify proteins and cellular structures, amplify oxidative damage and ultimately promote ferroptotic cell death (39, 40).

In ischemic stroke, this process is particularly relevant to neuronal injury because neuronal membranes are rich in PUFA-containing phospholipids and highly vulnerable to oxidative damage. Moreover, neurons have a high metabolic demand and relatively limited capacity to tolerate prolonged oxidative stress. Consequently, the combination of increased iron availability, enhanced PUFA-phospholipid peroxidation, and insufficient antioxidant defense can create a self-amplifying cycle of lipid oxidation and neuronal injury (32). Recent evidence further suggests that ferroptosis-associated lipid metabolic changes occur not only in neurons but also in astrocytes, microglia, and other glial cells, highlighting the importance of neuron–glia interactions in the progression of ferroptotic injury after ischemic stroke (41, 42).

### 3.3 Antioxidant defense systems

The accumulation of lipid peroxides is normally controlled by multiple endogenous antioxidant systems, which play a critical role in preventing ferroptosis. Among these, the system Xc^−^ (cystine/glutamate antiporter)/glutathione (GSH)/ GPX4 axis represents the major defense mechanism against lipid peroxidation (43). System Xc^−^, composed of solute carrier family 7 member 11 (SLC7A11) and solute carrier family 3 member 2 (SLC3A2), mediates the exchange of extracellular cystine for intracellular glutamate. Cystine is subsequently reduced to cysteine and used for GSH synthesis. GSH provides the reducing capacity required by GPX4 to convert potentially toxic phospholipid hydroperoxides into relatively harmless lipid alcohols (44, 45). Following ischemic stroke, oxidative stress, metabolic disturbance, and inflammatory signaling can impair this antioxidant system. Reduced SLC7A11 activity or GSH availability limits the cellular capacity to maintain GPX4 activity, while decreased GPX4 expression or activity directly compromises the removal of lipid hydroperoxides (46). Consequently, lipid ROS accumulate and interact with the already elevated LIP, substantially increasing the susceptibility of ischemic neurons to ferroptosis. Experimental studies have consistently reported alterations in SLC7A11 and GPX4 accompanied by increased lipid peroxidation and neuronal injury after cerebral ischemia, whereas restoration of this pathway can attenuate ferroptotic damage (47–49).

Nuclear factor erythroid 2-related factor 2 (NRF2) provides an additional layer of antioxidant protection. As a central regulator of cellular antioxidant responses, NRF2 can induce a broad range of genes involved in redox regulation, glutathione metabolism, iron homeostasis, and detoxification of reactive species. Activation of NRF2 therefore enhances the cellular capacity to counteract oxidative stress and suppress ferroptosis (50). In ischemic stroke, however, the intensity and duration of oxidative stress may exceed endogenous antioxidant capacity, resulting in insufficient NRF2-mediated protection. Experimental evidence suggests that enhancement of NRF2 signaling can increase antioxidant defense and attenuate ferroptotic neuronal injury, partly through preservation of the SLC7A11/GPX4 pathway (51–53). Heme oxygenase-1 (HO-1) is an important downstream target of NRF2 and participates in the regulation of oxidative stress and iron metabolism. By promoting heme degradation and generating metabolites with antioxidant properties, including biliverdin and bilirubin, HO-1 can reduce oxidative damage and lipid peroxidation under appropriate conditions (54). Activation of the NRF2/HO-1 pathway has been reported to attenuate ferroptotic injury and protect neurons in experimental ischemic stroke models (55, 56). However, the role of HO-1 in ferroptosis is context-dependent. Because heme degradation also releases free iron, excessive or sustained HO-1 activation may increase intracellular iron availability and potentially promote iron-dependent oxidative damage (57). Therefore, HO-1 may exert both antioxidant and iron-promoting effects depending on the severity and duration of ischemic injury and the capacity of the cell to sequester or export the released iron.

**Figure 2:**
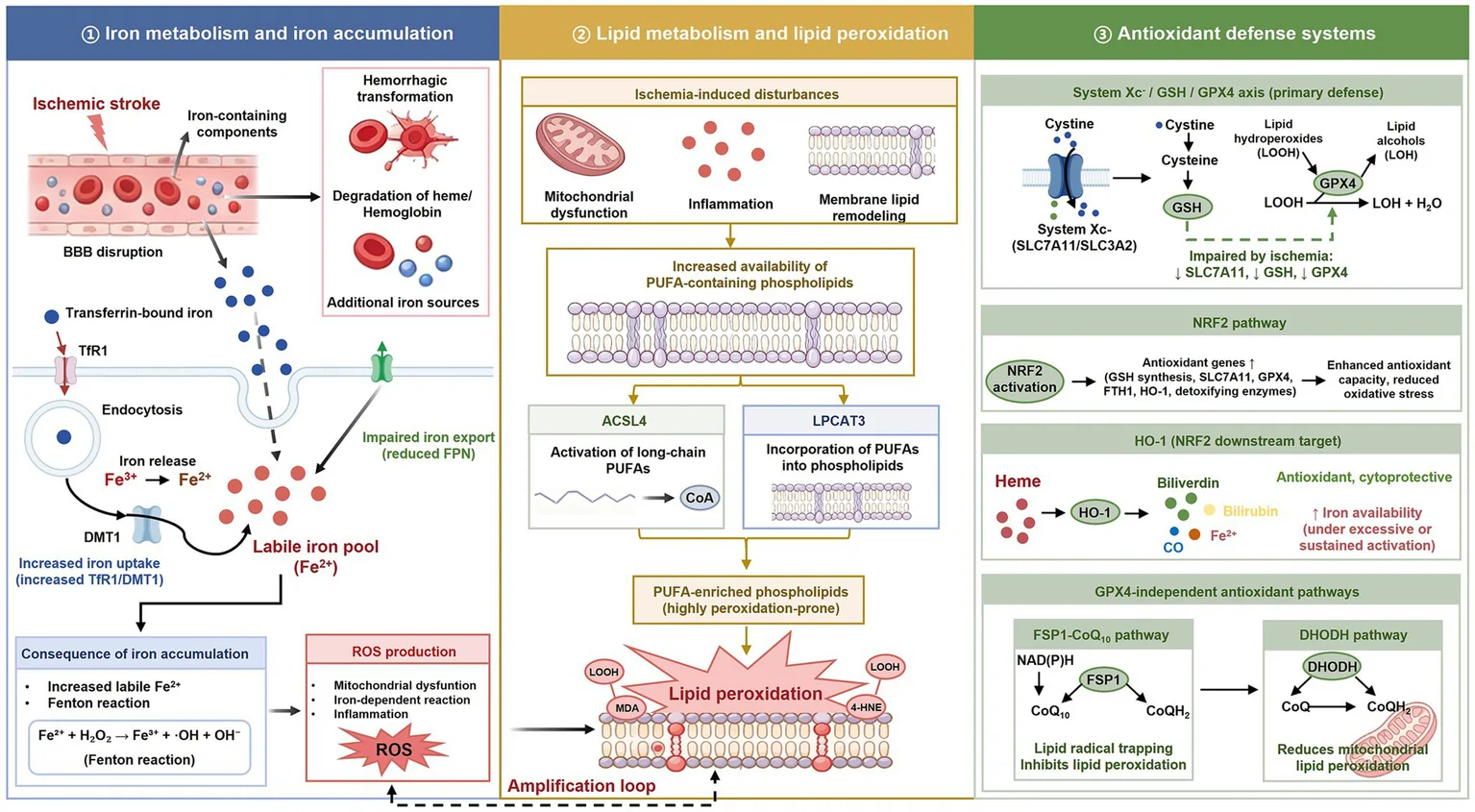
Molecular mechanisms of ferroptosis in ischemic stroke. Ischemic stroke promotes ferroptosis through iron accumulation, lipid peroxidation, and impaired antioxidant defense. The system Xc^−^/GSH/GPX4, NRF2/HO-1, FSP1-CoQ_10_, and DHODH pathways contribute to the regulation of ferroptotic susceptibility.

Ferroptosis is also regulated by antioxidant pathways that operate independently of GPX4. Ferroptosis suppressor protein 1 (FSP1) functions as a parallel defense system by using NAD(P)H to regenerate reduced coenzyme Q10 (CoQ10), or CoQH2, which acts as a lipid-soluble radical-trapping antioxidant and limits the propagation of lipid peroxidation. This pathway can suppress ferroptosis even when GPX4 activity is compromised, indicating that cellular resistance to ferroptosis is supported by multiple partially independent antioxidant mechanisms (58, 59). Similarly, mitochondrial dihydroorotate dehydrogenase (DHODH) can reduce mitochondrial lipid peroxidation through the CoQ/CoQH2 system, providing an additional layer of protection against ferroptotic damage (60) (Figure 2).

## 4 Context-dependent regulation of ferroptosis by HIF-1α after ischemic stroke

### 4.1 HIF-1α and iron homeostasis

HIF-1α can regulate ferroptosis by altering intracellular iron availability through multiple components of the iron metabolic network, including TfR1, DMT1, FPN, ferritin, hepcidin, and HO-1. Because these proteins collectively regulate iron uptake, export, and recycling, their modulation by HIF-1α can either increase or decrease the LIP, thereby influencing the susceptibility of ischemic cells to ferroptosis (61). TfR1 and DMT1 are important mediators of cellular iron uptake. HIF-1α has been reported to increase TfR1 expression under hypoxic or pathological conditions, enhancing iron uptake and potentially expanding the intracellular LIP (62). In experimental ischemic stroke, increased HIF-1α expression has been associated with upregulation of TfR1, increased cerebral iron accumulation, and enhanced oxidative damage and lipid peroxidation (63). These findings suggest that excessive HIF-1α activation may promote ferroptosis by increasing the availability of redox-active iron. However, HIF-1α may also exert an opposite effect on iron homeostasis. FPN is the major cellular iron-export protein, and its increased expression facilitates iron efflux and limits intracellular iron accumulation. Experimental evidence indicates that HIF-1α can increase FPN expression under certain conditions, thereby improving iron utilization and reducing ferroptotic susceptibility (64). Similarly, HIF-1α may influence systemic and local iron homeostasis through hepcidin, which regulates FPN stability and iron export. Suppression of hepcidin and preservation of FPN-mediated iron efflux may reduce intracellular iron accumulation and exert a protective effect against ferroptosis (65). Thus, the effects of HIF-1α on iron metabolism depend on the relative regulation of iron uptake and iron export rather than on a single iron-related target.

### 4.2 HIF-1α and antioxidant defense

In addition to regulating iron availability, HIF-1α influences ferroptosis by modulating cellular antioxidant capacity, ROS production, and metabolic adaptation. The SLC7A11/GSH/GPX4 axis represents one of the major targets through which HIF-1α may regulate ferroptosis. SLC7A11-mediated cystine uptake supports GSH synthesis, whereas GPX4 uses GSH to reduce lipid hydroperoxides and prevent the propagation of lipid peroxidation (66). Several experimental studies have suggested that HIF-1α activation can enhance SLC7A11 and GPX4 expression, increase cellular antioxidant capacity, and reduce ROS and lipid peroxide accumulation following ischemic injury. For example, recent experimental evidence indicates that activation of HIF-1α can protect against ischemic brain injury by promoting the HIF-1α/SLC7A11/GPX4 axis, whereas suppression of HIF-1α attenuates this protective effect (47, 67). However, HIF-1α may also promote ferroptosis under certain ischemic conditions. Recent studies suggest that HIF-1α activation may be associated with reduced GSH availability, increased accumulation of ROS and Fe^2+^, and enhanced lipid peroxidation following ischemia–reperfusion injury (68). These apparently contradictory findings suggest that the effects of HIF-1α may depend on the severity of hypoxia, duration of ischemia, timing of HIF-1α activation, and the metabolic state of the affected cells.

HIF-1α also interacts with the NRF2 pathway, which represents a broader regulator of antioxidant defense. NRF2 activation induces multiple genes involved in glutathione metabolism, ROS detoxification, iron storage, and cellular redox homeostasis (53). Through interaction with NRF2-dependent pathways, HIF-1α may enhance endogenous antioxidant capacity and reduce oxidative stress (69). In particular, the HIF-1α/NRF2/HO-1 axis may provide coordinated regulation of oxidative stress and iron metabolism, thereby influencing ferroptotic susceptibility (70). Nevertheless, as discussed above, the HO-1 response may have opposing consequences because enhanced heme degradation can simultaneously generate antioxidant metabolites and increase the availability of free iron (57). Importantly, HIF-1α can also influence lipid peroxidation more directly. In an experimental ischemic stroke model, HIF-1α suppressed ACSL4 expression during the early phase of ischemia, thereby reducing PUFA-related lipid peroxidation and protecting against ferroptotic neuronal injury. ACSL4 promotes the incorporation of PUFA into membrane phospholipids and increases ferroptosis sensitivity; therefore, its suppression by HIF-1α provides a direct mechanism linking the hypoxic response to lipid metabolism and ferroptosis (71). Notably, ACSL4 overexpression aggravated ischemic brain injury, whereas ACSL4 knockdown reduced neuronal ferroptosis and microglia-associated neuroinflammation (72).

**Figure 3:**
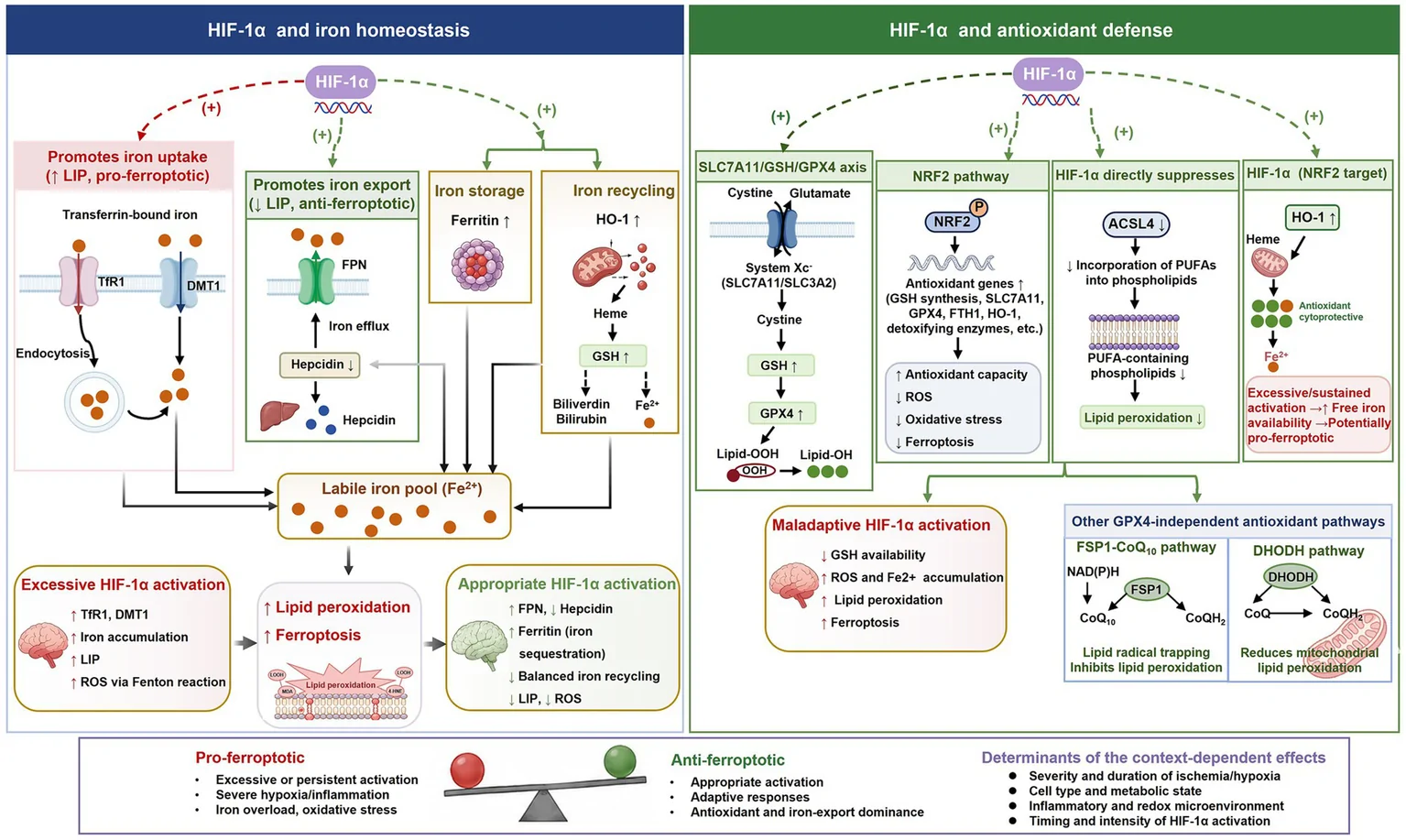
Context-dependent regulation of ferroptosis by HIF-1α after ischemic stroke. HIF-1α regulates ferroptosis through iron homeostasis, antioxidant defense, and lipid metabolism. Depending on the ischemic context, HIF-1α may exert either protective or pro-ferroptotic effects, with the net outcome determined by the balance between adaptive and maladaptive responses.

Overall, HIF-1α regulates ferroptosis after ischemic stroke through a complex network. These pathways can collectively reduce ROS accumulation and ferroptosis when HIF-1α activation promotes metabolic and antioxidant adaptation. Conversely, excessive or persistent HIF-1α activation may promote oxidative stress or iron accumulation under certain pathological conditions, thereby increasing ferroptotic susceptibility. Therefore, the ultimate effect of HIF-1α on ferroptosis is likely determined by the balance between its adaptive and maladaptive responses rather than by activation of any single downstream pathway (Figure 3).

## 5 HIF-1α-mediated neurovascular repair after stroke

### 5.1 Angiogenesis and vascular remodeling

Angiogenesis is a major component of post-stroke vascular repair and contributes to the restoration of blood supply in ischemic tissue. Following ischemic injury, hypoxia stabilizes HIF-1α, which subsequently activates the expression of angiogenic factors, particularly VEGF. VEGF promotes endothelial cell proliferation, migration, and survival and thereby stimulates the formation of new blood vessels in peri-infarct regions. Experimental and clinical studies have demonstrated that activation of the HIF-1α/VEGF axis is associated with enhanced post-stroke angiogenesis, reduced neuronal injury, and improved neurological recovery (73–75). VEGF exerts its angiogenic effects primarily through VEGF receptor 2 (VEGFR2) expressed on endothelial cells. Activation of the HIF-1α/VEGF/VEGFR2 signaling axis promotes endothelial cell proliferation and migration and facilitates the formation of new vascular networks (76). HIF-1α may also interact with other angiogenesis-related signaling pathways, including Notch signaling, to regulate endothelial cell behavior and vascular remodeling (77, 78). Thus, HIF-1α not only initiates the formation of new vessels but also contributes to the remodeling of the vascular network in response to ischemic injury.

### 5.2 Blood–brain barrier repair

Restoration of BBB integrity is an essential component of post-stroke neurovascular repair. Beyond promoting new vessel formation, effective vascular repair requires the maturation and stabilization of newly formed vessels, including restoration of endothelial tight junctions and interactions with pericytes and astrocytic end-feet. During acute ischemia, excessive HIF-1α activation can promote VEGF and matrix metalloproteinase-2 (MMP-2) signaling, leading to tight junction degradation and BBB disruption (19, 79). This response may be further aggravated by hyperglycemia, whereas endothelial HIF-1α inhibition alleviates BBB leakage and infarct injury in diabetic stroke models (80). These findings indicate that uncontrolled HIF-1α activation during the early stage may interfere with subsequent vascular stabilization and BBB repair. In contrast, appropriately regulated HIF-1α signaling may support BBB restoration during vascular remodeling. Under intermittent hypoxia, HIF-1α activation promotes VEGF-B expression, which counteracts VEGF-A-mediated endothelial barrier disruption and facilitates the maturation of newly formed vessels. This process is accompanied by restoration of endothelial zonula occludens-1 (ZO-1) expression, increased pericyte coverage, and recovery of astrocytic end-foot wrapping, ultimately contributing to the restoration of BBB integrity (81). Thus, HIF-1α may play a dual role in post-stroke BBB remodeling: excessive activation under severe acute ischemic conditions may impair barrier integrity, whereas appropriately regulated activation during the repair phase may promote vascular maturation and BBB stabilization.

### 5.3 Neurogenesis and neuronal remodeling

**Figure 4:**
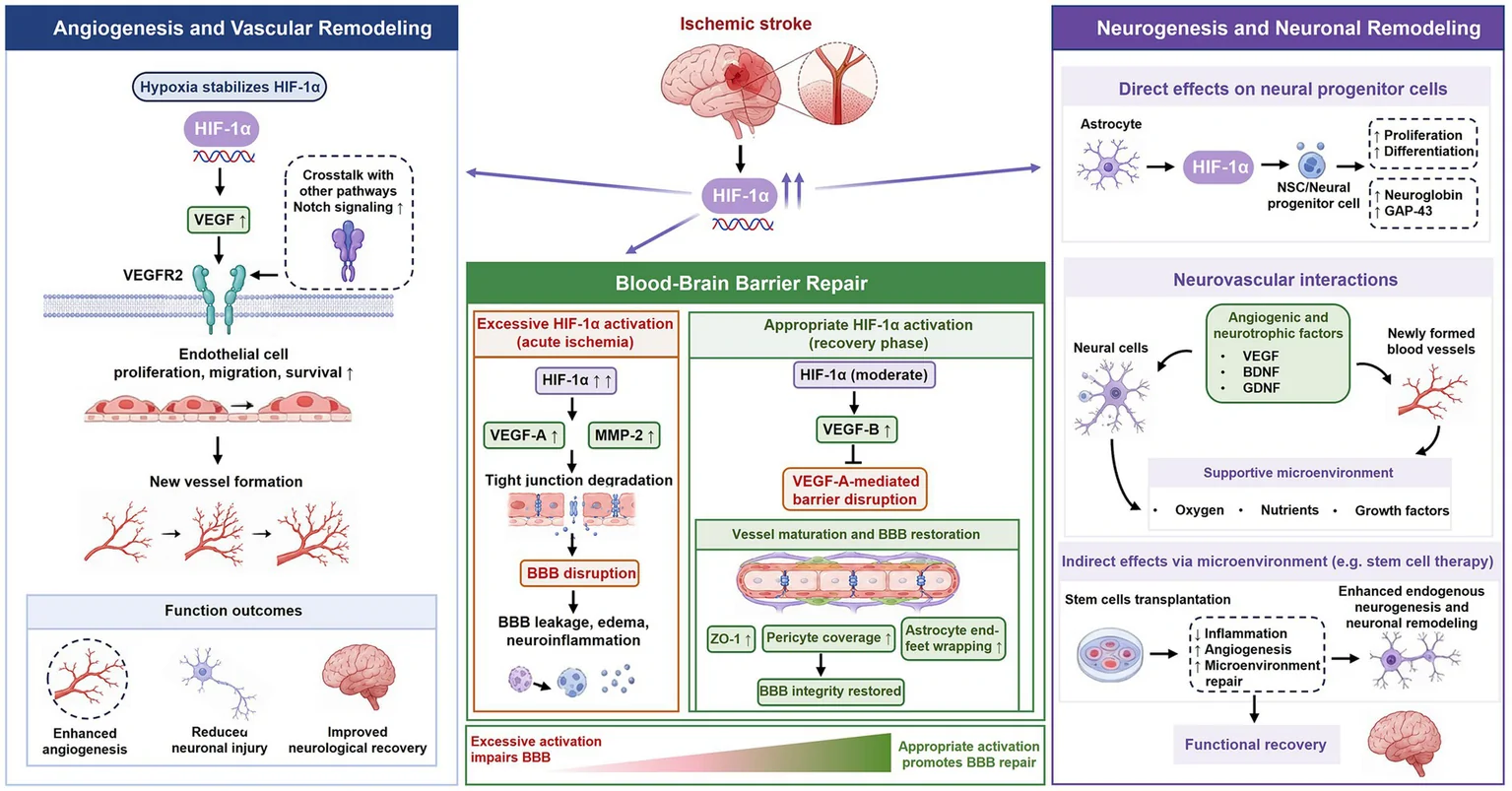
HIF-1α-mediated neurovascular repair after ischemic stroke. HIF-1α promotes post-stroke neurovascular repair through angiogenesis and vascular remodeling, blood–brain barrier restoration, and neurogenesis and neuronal remodeling. These processes interact to support tissue repair and functional recovery.

Neurogenesis and neuronal remodeling are important components of functional recovery after ischemic stroke. Following cerebral ischemia, NSCs and neural progenitor cells within endogenous neurogenic niches, particularly the subventricular zone and subgranular zone of the dentate gyrus, can be activated and contribute to the generation of new neurons (82, 83). HIF-1α, as a central regulator of the cellular response to hypoxia, may promote this process by regulating the proliferation and differentiation of neural progenitor cells and by modulating the surrounding vascular and metabolic microenvironment. Experimental studies have shown that activation of HIF-1α in astrocytes can influence NSC differentiation and increase the expression of neuroprotective and neuronal remodeling-related proteins, including neuroglobin and growth-associated protein 43 (GAP-43) (84). Given the important roles of GAP-43 in axonal growth, synaptic remodeling, and neuronal plasticity, these effects may contribute to the reconstruction of neural networks after stroke. The close interaction between neurogenesis and angiogenesis further highlights the role of HIF-1α in coordinating post-stroke tissue repair. Neural cells can release angiogenic and neurotrophic factors that support vascular remodeling, whereas newly formed vessels provide a supportive microenvironment for endogenous neural regeneration. Studies using stem cell-based approaches have further shown that short-term stem cell transplantation can remodel the ischemic microenvironment, reduce inflammation, and promote angiogenesis, thereby indirectly enhancing endogenous neural regeneration (85). Although these effects cannot be attributed exclusively to HIF-1α, HIF-1α-mediated hypoxic responses may contribute to the adaptation and survival of neural and vascular cells within the injured tissue. Thus, HIF-1α may promote post-stroke neurogenesis and neuronal remodeling through both direct regulation of neural progenitor cell responses and indirect effects mediated by vascular remodeling and improvement of the local regenerative microenvironment (Figure 4).

## 6 HIF-1α as a molecular link between ferroptosis and neurovascular repair

Ferroptosis can interfere with post-stroke neurovascular repair by promoting neuronal loss, endothelial injury, BBB disruption, and neuroinflammation. Excessive iron accumulation and lipid peroxidation not only directly damage neurons but may also impair endothelial cells and other components of the neurovascular unit. In addition, ferroptosis-associated inflammatory responses can further disrupt the local microenvironment and inhibit angiogenesis, neurogenesis, and vascular-neural interactions. Therefore, persistent ferroptosis may create an unfavorable environment for subsequent neurovascular remodeling and functional recovery. HIF-1α may function as a molecular coordinator linking ischemic injury to subsequent tissue adaptation and repair. On the one hand, HIF-1α can modulate iron homeostasis, antioxidant defense, and lipid peroxidation, thereby influencing the susceptibility of ischemic cells to ferroptosis. On the other hand, HIF-1α promotes angiogenesis, BBB repair, neurogenesis, and neurovascular unit remodeling. Importantly, these effects are highly context-dependent and may vary with the severity and duration of ischemia. Thus, HIF-1α may represent a molecular switch that integrates injury-associated signals with endogenous repair responses.

The dual role of HIF-1α suggests that therapeutic strategies targeting this pathway should consider the temporal evolution of ischemic stroke. During the early stage, excessive HIF-1α activation may contribute to iron accumulation, oxidative stress, and ferroptotic injury under severe ischemic conditions, and limiting these detrimental effects may be beneficial. During the recovery phase, however, HIF-1α-mediated antioxidant responses, angiogenesis, BBB repair, and neurogenesis may contribute to tissue restoration. Therefore, rather than simply activating or inhibiting HIF-1α, stage-, dose-, and context-dependent modulation of HIF-1α and its downstream pathways may provide a more rational therapeutic strategy for simultaneously limiting ferroptotic injury and promoting neurovascular repair after ischemic stroke.
